# Learning for a Robot: Deep Reinforcement Learning, Imitation Learning, Transfer Learning

**DOI:** 10.3390/s21041278

**Published:** 2021-02-11

**Authors:** Jiang Hua, Liangcai Zeng, Gongfa Li, Zhaojie Ju

**Affiliations:** 1 Key Laboratory of Metallurgical Equipment and Control Technology, Ministry of Education, Wuhan University of Science and Technology, Wuhan 430081, China; huajiang@wust.edu.cn (J.H.); zengliangcai@wust.edu.cn (L.Z.); ligongfa@wust.edu.cn (G.L.); 2 School of Computing, University of Portsmouth, Portsmouth 03801, UK

**Keywords:** dexterous manipulation, adaptive and robust control, deep reinforcement learning, imitation learning, transfer learning

## Abstract

Dexterous manipulation of the robot is an important part of realizing intelligence, but manipulators can only perform simple tasks such as sorting and packing in a structured environment. In view of the existing problem, this paper presents a state-of-the-art survey on an intelligent robot with the capability of autonomous deciding and learning. The paper first reviews the main achievements and research of the robot, which were mainly based on the breakthrough of automatic control and hardware in mechanics. With the evolution of artificial intelligence, many pieces of research have made further progresses in adaptive and robust control. The survey reveals that the latest research in deep learning and reinforcement learning has paved the way for highly complex tasks to be performed by robots. Furthermore, deep reinforcement learning, imitation learning, and transfer learning in robot control are discussed in detail. Finally, major achievements based on these methods are summarized and analyzed thoroughly, and future research challenges are proposed.

## 1. Introduction

The concept of robot gripping originated in 1962 with industrial robot Unimate which used a two-finger to grab wooden blocks and stack them together. The robot is designed to mimic the function of humans, so the pioneers of the field have done a lot of research on the grasp and manipulation mechanism. Human beings can manipulate objects and explore the world in various environments, so we also want robots to be as capable as humans. However, manipulation of the robot is not as simple as we think even though studies have been conducted for decades [1]. Although robotics has gained vast progress in mechanical design, perception, and robust control targeted to grasp and handle objects, robotic manipulation is still a poor proxy for human dexterity. To date, no robots can easily hand-wash dishes, button a shirt, or peel a potato.

Children are born with the ability to grab, and then get the adult-equivalent competence for planning sequences of manipulation skills after the learning of 9 years [2]. Neuroscience studies have shown that humans can grasp steadily and perform a variety of dexterous manipulations based on rich perceptual information and intelligence, so researchers want robots to have human-like abilities. Yaxu et al. analyze and compare existing human grasp taxonomies and synthesize them into a single new taxonomy [3]. Although a variety of research is carried out, how to implement various grasps and manipulation is still a problem of its own [4].

In order to realize the intelligent operation of the robot, it can be summarized into two main functional requirements, the first is the visual perception, the other is the intelligence of the robot. In the early stage, robots did not have the ability of perception. They grasped the robot mainly by means of manual teaching, hard coding, data gloves, and other tactile sensors. With the breakthrough of hardware technology, the integration of multi-model information such as vision, touch, and perception enables robots to identify the pose of the target more accurately [5]. So far, the biggest challenge at present is how to learn the optimal grasping strategy based on visual information.

At present, although the robot can perform some simple repetitive tasks well, it still cannot adapt to the complex environment with shielding or changing lighting conditions in real time. With the increasing demand for intelligent robots, it is urgent to design a robot grasping solution with independent ability of decision-making and learning. Therefore, the robot is a high-level embodiment of artificial intelligence in the physical world, and automation is the basis of intelligence [6]. The rapid development of artificial intelligence technology that encapsulates models of uncertainty further advances in adaptive and robust control. These machine learning algorithms for object grasps mainly include analytical and empirical approaches [7]. These methods are effective, but simplify the grasping environment and are based on hand-crafted features. Therefore, they are arduous, time-consuming, and cannot adapt to complex environments [8]. It is necessary to create a universal robotic solution for various environments, which have the ability to make decisions and learn independently. At present, deep reinforcement learning is the main method of intelligent decision and control of robots, which enables robots to learn a task from scratch. This method requires a lot of trials and incurs many errors, which is difficult to apply to actual robot manipulation [9]. To solve this problem, imitation learning and transfer learning are proposed. Ultimately, it is hoped that an end-to-end neural network can be constructed to output the motor control of each joint simply by inputting the observed image [10].

To sum up, this paper will present a state-of-the-art survey on an intelligent robot with the capability of autonomous deciding and learning. The paper first reviews the main achievements and research in adaptive and robust control. The survey reveals that the latest research in deep learning and reinforcement learning has paved the way for highly complex tasks to be performed by robots. Furthermore, three main methods of deep reinforcement learning, imitation learning, and transfer learning are discussed for a robot. Finally, major achievements based on these methods are summarized and analyzed thoroughly, and future research challenges are proposed.

The remainder of the paper is arranged as follows. In Section 2, we survey the theory of how to form stable manipulation and introduce the research background. Section 3 focuses on how a robot can learn a motor control policy via deep reinforcement learning as a complete solution to a task. Section 4 describes approaches of imitation learning to master skills by observing movements from only a small number of samples. Section 5 describes approaches that knowledge can be transferred to the real robot by building a robot virtual simulation system based on transfer learning. Finally, latest applications and future research directions are discussed.

## 2. The Background

**Figure 1:**
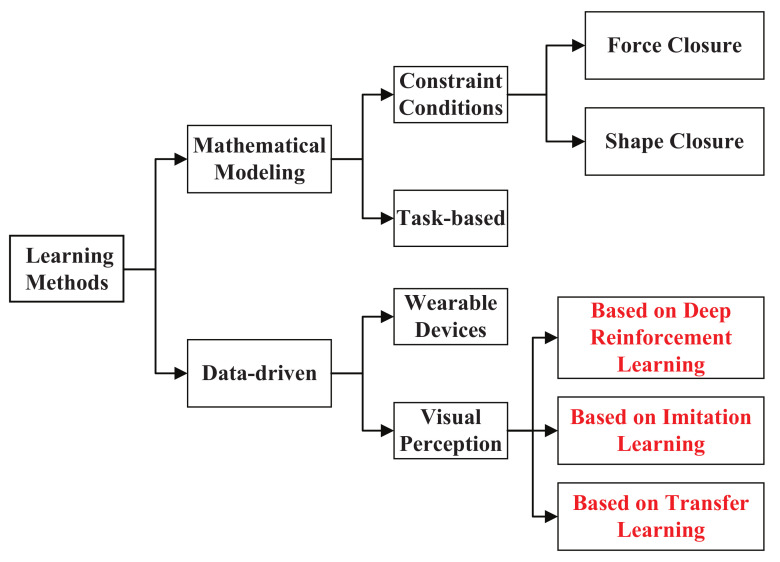
Overview of the structure of the survey.

For decades, researchers have worked to establish the theory of how to form a stable manipulation. However, manipulating an object is a far more daunting problem. At present there are mainly two directions, one way is to set up a mathematical model aimed at determining the minimal number and optimal positions of the fingertips on the object‘s surface to ensure stability [11]. The second way is data-driven methods by establishing a database about the manual grasping type, the optimal solution of grasping can be obtained by analyzing and understanding the data with sensors information and prior knowledge [12]. The survey is structured as Figure 1.

The method of mathematical modeling needs to take many constraints into consideration and obtains the optimal value by establishing the objective function [13]. As shown in the Figure 1, the closed conditions are the major factors to be considered. Force closure and shape closure are two important manifestations of closed conditions, which are widely used in the plan of manipulation [14]. Force closure means that the contact force spiral on the surface of the object is in equilibrium with the external force spiral. Shape closure is a stronger constraint than force closure, but it increases the complexity of calculation and the difficulty of control accordingly [15]. Therefore, grasping stability is evaluated by force closure in most cases. Another scheme of mathematical modeling is that establishing an extremely suitable policy based on specific tasks [16]. For instance, a statistical model of interference distribution based on the grasping task was proposed, so the optimal grasping pose for the specified task can be obtained [17]. This solution greatly reduces the complexity of manipulation and improves the efficiency of policy planning. Yet these methods of mathematical modeling have to rely on the accurate geometric model of the target, so they are difficult to meet the actual need [18]. Moreover, the consumption of optimizing the objective function is very large and cannot ensure the real-time update of robot systems.

With the advancement of hardware and machine learning technology, data-driven methods that can reduce the complexity of the computation without listing all possibilities are widely used in robot manipulation [11]. The ability of perception and understanding are improved via feature recognition and classification, then the probability model of manipulation can be learned to perform the task [19]. Nowadays, there are two main solutions for data-driven methods. One scheme is that delivering body information of manipulation to the robot via some wearable sensing devices, and the other is extracting object features to plan the policy based on visual perception [20]—collecting the data via wearable devices, and analyzing the coordinated movement relationship among multiple joints of the human hand [21]. Then the features of the manipulation pose can be extracted, so as to establish the mapping between the human hand and the dexterous manipulator [22]. This scheme can explore the deep mechanism of human hand, simplify the space dimension of the robot manipulation, and provide a theoretical basis for human–machine collaboration [23].

Currently, learning to manipulate objects based on the scheme of visual perception has been a research focus of data-driven methods [24]. The method of extracting features from images provides a new direction for learning robot manipulation, but traditional methods of feature extraction mainly rely on the prior knowledge, so merely part of the information can be utilized effectively [22]. Owing to the great breakthrough of deep learning, the robot can extract more generalized features autonomously[25]. Due to the excellent capability of feature extraction, the deep learning network has achieved fantastic results in machine perception and image processing [26]. At the same time, deep learning can also be combined with the method of mathematical modeling to learn the robot manipulation, but the biggest shortcoming is still the lack of the entire system model [27]. Therefore, deep reinforcement learning (DRL) is proposed to realize the end-to-end learning from perception to robot manipulation.

However, it is difficult for agents to ensure the effectiveness of deep reinforcement learning in complex scenarios due to the limitations of sparse rewards. There, researchers put forward the idea of hierarchy according to the characteristics of human intelligence [28]. Hierarchical deep reinforcement learning can decompose the whole task, and then implement it step by step by lower levels of policy. According to the latest research in recent years, it is found that the effect of hierarchical deep reinforcement learning is far better than previous algorithms, which can not only adapt to complex problems, but also solve the problem of sparse rewards [29].

Reinforcement learning enables the robot to interact with the environment through trial and error, then the optimal strategy can be learned by maximizing the total return [30]. The method of deep reinforcement learning require a large number of samples and trials, so they are feasible for the field of image recognition but hardly suit for real robot manipulation. Nowadays, there are two ways forward to solve this problem [31]. One is imitation learning, in which machines can quickly learn to manipulate by observing a demonstration or a small amount of data. The method can reduce the complexity of robot strategy space and improve the learning efficiency [32]. The other one is transfer learning, in which the robot firstly learns to manipulate in the simulation environment, and then transfer the knowledge to the real. During the training of the real robot, valuable information is extracted from the simulated neural network, which greatly accelerates and strengthens the learning effect [33]. These three methods of robot learning will be described and analyzed in detail in this paper.

## 3. Deep Reinforcement Learning

Traditional manipulation learning methods need to know the model of the whole system in advance, but it is impossible in most cases in practice. Therefore, the method of reinforcement learning is inevitable, which enables the robot to make policy independently [34]. The traditional algorithm of reinforcement learning is the dynamic planning that deals with finite state space—then the optimal strategy can be obtained based on the accurate model, but it cannot solve the problem of robot manipulation. Therefore, deep reinforcement learning independent of the dynamic model that can adapt to the environment well is proposed to handle the task of continuous state space [35]. Deep reinforcement learning combines the perception ability of deep learning and the decision-making ability of reinforcement learning, which can learn the actions of the robot directly from images. Nowadays, deep reinforcement learning has become a key research direction in the field of robotics. Markov decision process (MDP) is the basis of reinforcement learning, the function of action-state value can be obtained from the expected sum of rewards [36]. The formula of value function is shown as Formula (1).

(1) $$Q_{\pi }\left(s,a\right)=E_{\pi }\left[\sum_{t=0}^{T}\gamma^{t}r_{t}|s_{t}=s,a_{t}=a\right]$$

**Figure 2:**
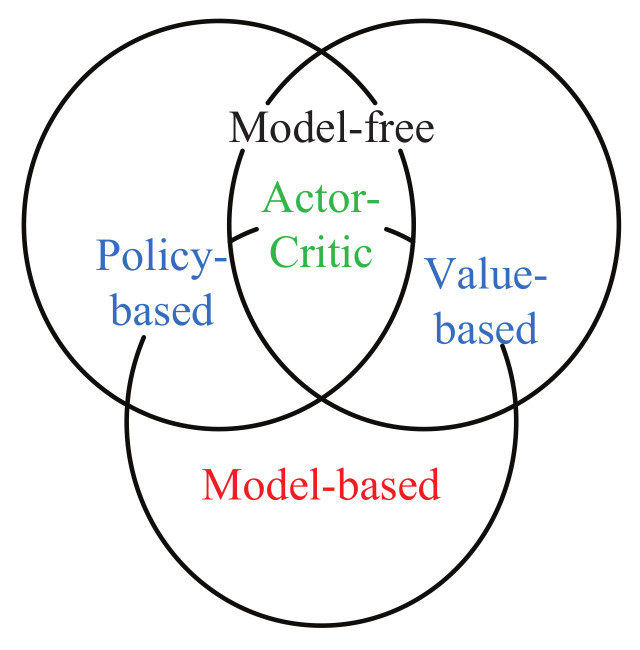
The classification of reinforcement learning.

In the formula, the expected sum of discounted rewards is defined as the function of action state value $Q_{\pi }\left(s,a\right)$. $E_{\pi }$ represents the expected value in the case of motion strategy π, $r_{t}$ represents the reward value for the corresponding moment, and $\gamma^{t}$ represents the discount factor. On the basis of whether the state transition probability and return are known, reinforcement learning also can be sorted into model-based and mode-free methods as shown in Figure 2. Model-based methods can generate an environment model via sample data. Model-free reinforcement learning algorithms do not need to model the environment, but interact directly with the environment to learn relevant strategies. These two types of reinforcement learning algorithms can be divided into two categories based on the solution approach: the value-based learning method and the policy-based learning method [37]. At the same time, these two methods also can be combined to get a new method, actor-critic. This section will introduce representative algorithms of deep reinforcement learning in the field of robot manipulation.

### 3.1. Model-Based Methods

The model-based method of deep reinforcement learning can construct a dynamic probabilistic model via lots of data, and learn the best strategy from the value function of sate [38]. At the same time, methods can avoid interaction with the environment and train the strategy based on learned dynamic models. Therefore, the prior knowledge is an advantage of the model-based approach. Therefore, the development of predictive models based on prior knowledge of tasks and environment is the focus of subsequent research. The optimal solution can be obtained by the algorithm of value iteration and the algorithm of policy iteration when the model is known [39].

Some researches of robot manipulation via value-based deep reinforcement learning can be found. Todd et al. enabled the robot to play football via the state transition probability model of decision tree (DT) [40]. Rudolf et al. build a state transfer probability model based on the local linear system estimation (LLSE). The method to gain the value function is converted into a problem of solving linear programming that enables the two-link mechanical arm to play table tennis [41]. Connor et al. built the model of manipulation based on the convolutional neural network (CNN) and the mechanical arm can dig beans [42]. Methods of value function can adjust the strategy in time with the state value, which greatly reduces the time of iteration.

Learning the optimal strategy by policy improvement and policy evaluation is the core of policy iteration [43]. The sum of expected rewards is calculated in the stage of policy evaluation and the stage of policy improvement is used to optimize the strategy via the result of policy evaluation. These algorithms work by perturbing the policy parameters in many different ways, and then moving in the direction of good performance [44]. Jan et al. trained the manipulation skill of hitting the baseball via combining the policy gradient with the motor primitive [45]. Gen et al. learned the walking skill of a bipedal robot based on the policy gradient [46]. Marc et al. proposed a model-based algorithm of probabilistic inference for learning control (PILCO) for robot grasping, which incorporated the image information provided and the spatial constraints of manipulation into the learning process [47]. Currently, mainstream methods of policy iteration include Guided Policy Search (GPS) [48] and Cross-entropy method (CEM) [49].

The GPS proposed by Sergey Levine is a representative example of robot control achieved by combining traditional control algorithms with deep learning [50]. By the traditional control algorithm to create an end-to-end neural network, the tasks such as hanging clothes and opening bottle caps can be completed autonomously. Feature points are outputted through a convolutional neural network and in series with the basic parameters, and then the motor torques are output from two fully connected layers. Mechanical arm model information and precise information of the door can generate an optimal trajectory by traditional robot control algorithms such as linear quadratic regulator. The trained neural network can optimize the control trajectory based on these samples, and then explore the state and action space. However, the efficiency of the traditional method is very low, so the method of CEM is proposed to take samples and gain the probability of picking up the object [51].

The algorithms of policy iteration can be used to initialize the parameters with expert knowledge and accelerate the convergence process of strategy optimization. They are easy to implement and work very well for policies with a small number of parameters. Model-based methods of reinforcement learning can greatly improve the utilization of data and effectively reduce the cost of learning [52].

### 3.2. Model-Free Methods

The model-based methods can approximate the current value via the previous state value function, but not suitable for robot manipulation that accurate kinetic models are difficult to build. Therefore, model-free methods will be the focus of research in which agents interact with the environment via trial and error to gradually optimize the strategy [53]. At present, there are mainly two research directions, methods of value-based and policy-based. The representative algorithm based on value function is Q-learning in which the selection policy of action is greedy [54]. The algorithm updates the action-value function in accordance with the following formula:

(2) $$Q\left(s,a\right)=Q\left(s,a\right)+\alpha \left[r+\gamma \max Q\left(s^{\prime },a^{\prime }\right)-Q\left(s,a\right)\right]\;$$

Minoru et al. adopted the Q-leaning algorithm to realize the robot hitting the ball to the designed position based on visual enhancement [55]. Q is a tabular solution to evaluate the quality of each action. However, most scenes of robot control have so huge a state-space or action-space that the cost of using Q table is a big consumption. The method of function approximation is the solution to upper problem, which can be expressed by the function of linear or nonlinear [56]. Therefore, deep Q-network (DQN) that is combined of Q-learning and deep neural network is proposed to explore the high-dimensional space [57]. Zhang et al. train the grasping strategy of a three-joint robot based on DQN. Due to the difference between the simulation environment and real scene, the grasping effect of the controller is not good enough [58]. In order to perform dexterous manipulation of the robot, the improved algorithm of DQN was proposed [59].

Value-based methods cannot enumerate the quality of every action in continuous action-space, so it is impossible to calculate the optimal value. Therefore, another more direct way is needed to solve this problem, namely the policy gradient. Policy-based methods can directly parameterize the strategy and optimize the parameters based on the evaluation function [60]. The estimation of value function is still needed in the policy-based method, but the difference lies in whether the final strategy is directly measured by parameters or derived from the value function [61]. The policy-based algorithm could solve the problem of high cost in a real scenario and generate guided training samples by optimizing the trajectory distribution [62]. Schulman et al. proposed the algorithm of trust region policy optimization (TRPO), which updated policy parameters by optimizing the objective function [63]. Then the improved algorithm of proximal policy optimization (PPO) achieved a better result than TRPO when learning robot manipulation in the virtual simulation environment [33]. Mirowski et al. came up with that the agent learned to navigate in a complex environment based on the algorithm of asynchronous advantage actor-critic (A3C) [64]. In addition, Levine et al. learned the robot manipulation skills by optimizing the parameterized strategy based on various methods of policy gradient [65].

Policy gradient can select the appropriate strategy from continuous actions, but it can only be updated at the end of the round. Therefore, the algorithm of actor-critic was proposed which combined the advantages of value-based methods and policy-based methods. Lillicrap et al. proposed an algorithm of deep deterministic policy gradient (DDPG) based on the actor-critic framework, and realized robot manipulation in the simulation environment [66]. However, the algorithm of DDPG needed to train two networks, so the normalized advantage function (NAF) of one network was proposed that applied the algorithm of Q-learning into continuous action space [67]. Gu et al. proposed an algorithm of asynchronous NAF that had a trainer thread and multiple collector threads, in which the latest parameters of neural network were continuously shared with each robot [68]. The above achievements indicate that trained predictive models can be used by real robotic systems to manipulate unseen tasks in the past.

To conclude, motion planning of the robot is a tedious and complex task, so traditional algorithms of reinforcement learning cannot fulfill the task of high degree of freedom in continuous action space. If it is in discrete action space, the method of DQN can achieve high-performance. The method of DDPG can solve the tasks of continuous space and low action dimension. The algorithm of A3C is recommended when the action dimension is high and data are easy to obtain.

For more complex tasks, a stable and efficient algorithm of soft actor-critic (SAC) is proposed for real-world robot learning [69]. What is more, the algorithm of SAC can perform robotic tasks in a matter of hours and work in a variety of environments using the same set of hyperparameters. By comparison, the policy-based approach can more easily integrate the expert knowledge to accelerate the convergence process of the strategy. At the same time, policy-based methods has fewer parameters than value-based methods, so the learning efficiency is higher. The strategy obtained from the model-based algorithm of deep reinforcement learning depends on the accuracy of the model, while the model-free algorithm can improve the robustness of the learned strategy by a large number of interactions with the environment. Therefore, model-free methods can learn more generalized strategies. Various methods of deep reinforcement learning have their own advantages and disadvantages. It is necessary to make a trade-off among computational complexity, sample complexity, and strategy performance. Therefore, the effective combination of the advantages of various methods of deep reinforcement learning is the current research focus for improving the performance of robot manipulation. The characteristics of robot algorithms based on reinforcement learning are summarized in Table 1.

It can be seen from the above research that deep reinforcement learning can successfully enable robots to master task skills through learning. The method will become the most promising way to realize a universal robot. However, methods based on deep reinforcement learning have the disadvantages of slow convergence and long computation time in the field of robot learning. It is a great challenge to perfectly match the rewards with a series of actions and achieve the rapid convergence of the entire network. In order to solve the problem of high consumption in training data and cost, the method of imitation learning has been further explored.

## 4. Imitation Learning

**Figure 3:**
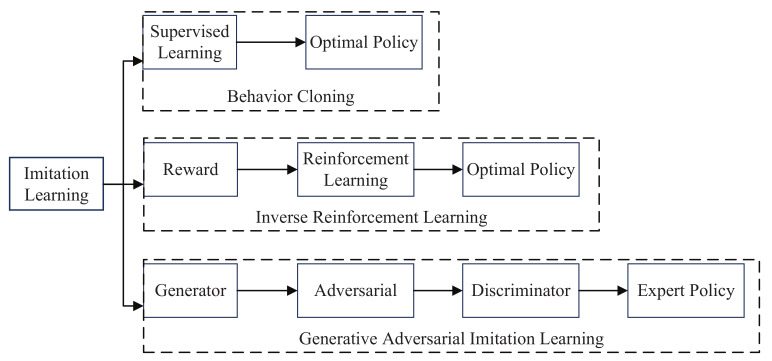
Classification of imitation learning.

Imitation learning, in which the robot learns manipulation by observing the expert’s demonstration, and skills can be generalized to other unseen scenarios. This process not only extracts information of the behavior and surrounding environment, but also learns the mapping between the observation and the performance. The task of robot manipulation can be viewed as a Markov decision process, then encoding action sequence of the expert into state-action pairs that are consistent with the expert. Imitation learning can train data from good samples instead of learning from scratch, so the learning efficiency is further improved [70]. By combining with reinforcement learning mechanisms, the speed and accuracy of imitation learning can be improved. Currently, the methods of imitation learning can be divided into behavior cloning (BC), inverse reinforcement learning (IRL), and generative adversarial imitation learning (GAIL) [71]. The classification of imitation learning can be seen in Figure 3.

### 4.1. Behavior Cloning

The essence of BC is direct policy learning, which enables the distribution of state-action trajectory generated by the agent to match the given teaching trajectory [72]. The traditional method of behavioral cloning is that the robotic arm learns the trajectory of the movement by manual guidance or teaching box. However, it can only simply repeat learned motions, not adapt to environmental changes. With the development of statistical learning, methods of machine learning have been introduced to identify basic units of robot manipulation. Takeda et al. trained a robot manipulation policy that can dance with humans based on hidden Markov model (HMM) [73]. However, such methods describe the trajectory through a series of discrete states and transitions between states, which does not allow for continuous smooth path and direct control of the robot motion. In order to solve the related problems, Calinon et al. enabled the robot to complete a series of operations from simple to complex based on the Gaussian mixture model (GMM) and Gaussian mixture regression (GMR) [74,75]. Multiple Gaussian distributions are used to model different stages of the trajectory and the covariance can be used to describe the uncertainty.

Gams et al. proposed dynamic motion primitives (DMPs) to generate a stable and generalizable strategy based on trajectories [76]. Methods of DMPs can generate trajectory of arbitrary complexity that can be used to describe robot manipulation. The disadvantage of DMPs is the need of a deterministic model, yet the fact that demonstrations cannot be completely alike. Therefore, it is difficult for this method to model the uncertainty of multiple demonstrations, resulting in a poor fit of the system as a whole. Zhang et al. proposed a virtual reality teleoperation system to collect high-quality demonstrations of robot manipulation, then the control strategy can be obtained via visuomotor learning (VL) [77]. The result shows that imitation learning can be surprisingly effective in learning deep policy that map directly from pixel values to actions, only with a small amount of learning data.

However, the problem of BC is that the number of samples is not enough, so the agent cannot learn situations that are not included in samples. Therefore, in the case of a small amount of samples, the strategy obtained by behavioral cloning is not generalizable. In order to solve the learning problem of insufficient samples, the method of inverse reinforcement learning is proposed [78].

### 4.2. Inverse Reinforcement Learning

Inverse reinforcement learning is a method of evaluating how well an action is performed via reward function, which is an abstract description of behavior. Compared to methods of behavioral cloning, IRL is an efficient paradigm of imitation learning that is more adaptable in responding to different environments. When the execution environment or robot model changes significantly, the resulting mapping function will be difficult to apply and will need to learn again [79]. Whereas the method of IRL is more task-related, the appropriate strategy can be obtained based on the previous reward function after receiving new information from the environment and the model [80]. Inverse reinforcement learning can be classified according to the algorithms it depends on.

Abbeel et al. proposed the max-margin principle (MP) of obtaining a reward function based on teaching data, in which the difference between the optimal strategy and other suboptimal strategies can be maximized [81]. Ratliff et al. suggested the framework of maximum marginal planning (MMP) based on the principle of maximum margin, and transformed the learning of reward function into a structural prediction [82]. The method of maximum marginal programming is very expensive to solve the MDP, so Klein et al. proposed a method of structured classification (SC) to learn the reward function without solving MDP [83]. Ho et al. proposed a neural network on the basis of apprenticeship learning (AL), and updated it via the method of policy gradient. It is hard to determine the quality of actions in actual scenarios [84]. The above methods are all artificial design features of the reward function that are difficult to generalize to the high dimensional and continuous robot state space. Therefore, Xia et al. proposed the neural inverse reinforcement learning (NIRL), which is still based on the framework of maximum margin [85].

The disadvantage of maximum margin methods is that different reward functions will lead to the same expert strategy in many cases, thus resulting in ambiguity. Therefore, many algorithms of inverse reinforcement learning are proposed based on the probabilistic model to overcome this problem [86]. Ziebart et al. constructed a probabilistic model for sequential policy by maximum entropy inverse reinforcement learning, which can ensure the manipulation strategy has better performance when the teaching data are not optimal and the reward function is random deviation [87]. Finn et al. updated the policy based on the maximum entropy IRL and constructed reward function to help training via expert data [88]. The method of inverse reinforcement learning based on maximum entropy needs to know the state transition probability of the system. Therefore, Boularias et al. established the maximum relative entropy model to solve the model-free problem [89]. Peng et al. presented a data-driven deep reinforcement learning framework to train humanoid robots in virtual environment via the algorithm of DeepMimic and then learned a series of difficult manipulation skills [90]. The resulting strategy is highly robust and the generated natural motion is almost indistinguishable from the original motion capture data in the absence of perturbations.

Methods of behavioral cloning and IRL learn strategies from demonstrations, but can not interact with the expert to further optimize the policy [91]. Therefore, the method of generative adversarial imitation learning is proposed to solve the problem based on adversarial networks [92].

### 4.3. Generative Adversarial Imitation Learning

The method of GAIL is implemented by comparing the difference between the generated strategy and the expert strategy. Iterative confrontation training can be performed to make the distribution between the expert and the agent as close as possible [93]. Generative adversarial networks (GANs) have been successfully applied to policy imitation problems under model-free settings. Baram et al. proposed the algorithm of model-based generative adversarial imitation learning (MGAI) based on a forward model to make the calculations completely divisible, which allows the use of accurate discriminator gradients to train strategies [94]. The use of pure learning methods with simple reward functions often results in non-human and too rigid movement behaviors. Merel et al. extended the algorithm of GAIL that the training of general neural network strategies can generate human-like motion patterns from limited demonstrations without access to actions. This method constructs strategies and shows that they can be reused to solve tasks when controlled by a higher-level controller [95]. They are vulnerable to cascading failures when the agent trajectory diverges from the demonstrations. Wang et al. added a variation auto-encoder (VAE) to learn semantic policy embeddings that made the algorithm of GAIL more robust than the supervised controller especially with few demonstrations. Leveraging these policies, a new version of GAIL can be developed to avoid mode collapse and capture many different behaviors [96].

Unfortunately, methods of imitation learning tend to require that demonstrations are supplied in the first-person that is limited by the relatively hard problem of collecting first-person demonstrations. Stadie et al. presented a method of unsupervised third-person imitation learning (TPIL) to train agent to correctly achieve goal in a simple environment when the demonstration is provided from a different viewpoint [97]. Standard imitation learning methods assume received examples that could be provided in advance, which stands in contrast to how humans and animals imitate. Liu et al. proposed an learning method of imitation from observation (IFO) based on video prediction with context translation, which ensured output of different domains consistent [98]. The assumption in imitation learning is lifted that shows the effectiveness of our approach in learning a wide range of real-world robot manipulation.

The way that robots learn desired strategies based on deep reinforcement learning in real scenario will face the problem of large data requirement, high cost of trial and error and long training process. To enable learning of robot manipulation, roboticists focused their efforts on imitation learning that coincided with the learning process of human. Methods of imitation learning combined expert demonstrations with appropriate algorithms of machine learning, which can provide a simple and intuitive framework of robot learning and reduce the cost of deployment. Therefore, imitation learning is an effective method for the system to obtain control strategies when an explicit reward function is insufficient, using supervision provided as demonstrations of the expert.

Although the method of BC is intuitive and simple to implement, a large amount of data is required and the learned policy cannot adapt to the new environment. Although the method of IRL makes up for shortcomings of above situations, the consumption of training time is still costly. The method of GAIL introduces the idea of generative adversarial networks for imitation learning, which has better performance than the other two methods in high-dimensional situations. A major drawback of GAIL is the problem of model collapse because the diversity of generated images is often smaller than the real data. To summarize, imitation learning has been a key method in the field of robot manipulation. Current algorithms solve the problem of designing the reward function to a certain extent and accelerate the rate of learning by initializing strategies based on teaching data. Robot algorithms based on imitation learning are summarized in Table 2. However, there are still some problems in imitation learning, such as the high consumption of collecting data and the local optimal solution of policy, which may lead to the poor effect of learning. Therefore, some scholars have put forward the method of transfer learning, in which the model of learning is trained in a simulation environment and then the knowledge is transferred to the real robot, so as to acquire the skills of robot manipulation more efficiently.

## 5. Transfer Learning

**Figure 4:**
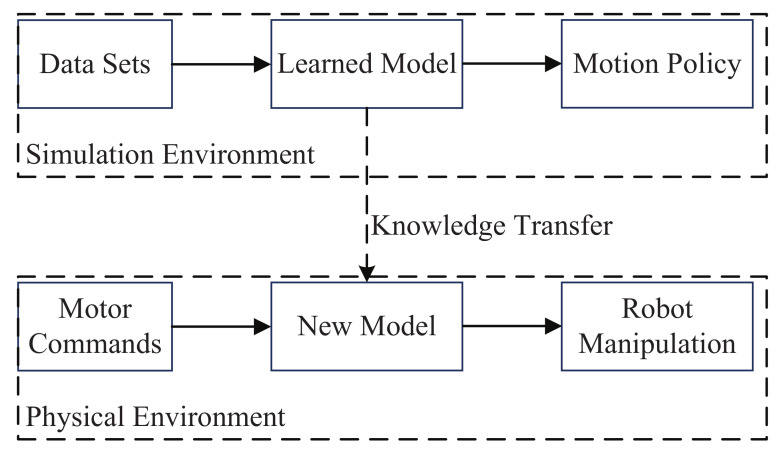
Principle of transfer learning for robot manipulation.

Robot manipulation is so complex that the consumption of obtaining an optimal solution is costly. Obtained policy based on deep reinforcement learning can only be applied in one task and have to start from scratch whenever the environment changes slightly. By introducing transfer learning into robot deep reinforcement learning, the data in the simulated environment can be used to help the robot better learn control strategies (Figure 4). The method of transfer learning with learning ability can divert knowledge from the source task to the target task by sharing learned parameters with the new model [99]. This optimization method of transfer learning can greatly improve the generalization of the original model and the speed of modeling new tasks. Data sets of the task which include position, velocity, and force are collected and then used to learn the skill model. Then the knowledge of the learned model can be transferred into real robot so as to obtain the new model that can reproduce the robot manipulation in new environments [100].

However, it is not easy for robots to transfer and learn, because there is a reality gap between the simulation and reality. The policy will not adapt to changes in the external environment if trained in a flawed simulation. In addition, the physics of sliding friction and contact forces also cannot be perfectly simulated [101]. Several improved methods of transfer learning are proposed that will be elaborated briefly in this section.

### 5.1. Better Simulation

For many robot manipulations, data sets of real world are costly to obtain, but easy to collect in the simulation environment. Tzeng et al. proposed a novel method of domain adaptation for robot perception without expensive manual data annotation before policy search [102]. The improved method of transfer learning compensates for domain shift more effectively than previous techniques by using weakly paired images. Zhu built a highly similar simulation framework named AI2-THOR in which the optimal strategy was trained in high-quality 3D scenes [103]. Agents can manipulate and interact with objects in the framework, so a huge number of samples are collected. At the same time, the method is end-to-end trainable and converges faster than other methods. In the robot simulation environment, only limited parameters can be used to simulate the physical environment, so there are errors compared with the real situation. Peng et al. proposed a recurrent neural network to reduce the gap between virtual and real, which improved the effect of robot transfer learning by narrowing the training error [104]. The neural-augmented simulation can improve the effect of robot transfer learning by narrowing the training error.

According to the above analysis, these methods can construct a better simulation environment, but they are all measured in definite states and actions to train the agent. Another idea is that highly adaptable strategy can be trained through randomized processing of states and actions. Then the system of the robot will respond to dynamic changes effectively in the real world without adjustments.

### 5.2. Policy Randomization

Although the simulation environments provide an abundant source of data and reduce the potential safety concerns during the training process, policies that are successful in simulation may not transfer to real world because of modeling errors. Algorithms of policy gradient are very effective in solving the high-dimensional sequential task of robot manipulation. Ammar et al. proposed a method of multi-task policy gradient to learn policy, which can transfer knowledge between tasks to improve learning efficiency [105]. The realization of end-to-end pixel-driven control for complex robot manipulation is an unresolved problem. Rusu et al. proposed progressive networks, which are a general framework that can reuse everything from the low-level visual functions to the high-level strategies. The speed of each robot joint can be obtained by input image only, which further verifies the feasibility of progressive neural networks [106]. Peng et al. proposed that dynamic and highly adaptive strategies could be obtained by randomizing the dynamics of the simulator during training, which can adapt to the significantly different situation [90].

Both above approaches have done extensive processing of the virtual environment to improve the performance of the simulator. However, none of studies can guarantee the adaptive capability required for real-world robots. The approach presented below is a higher-level complementary approach of enhanced transfer learning that produces policies which generalize across tasks.

### 5.3. Robust Policy

Methods of transfer learning have difficulty in obtaining policies which can generalize across tasks, despite collecting a large amount of data. He et al. proposed an attempt to learn a robust policy directly on a real robot based on model-predictive control (MPC), adapting to unseen tasks [107]. A continuous parameterization and policy can be learned simultaneously in simulation instead of end-to-end learning policy for single task. Then the multi-skill policy can be transferred directly to a real robot that is actuated by choosing sequences of skill latents. The model of MPC is composed of the pre-trained policy executed in the simulation, run in parallel with the real robot. Agents trained in the simulator may not be invalid in the real world when performing actions due to the gap between training and execution environments. Ramakrishnan proposed the oracle feedback to learn a predictive model of blind spots in order to reduce costly errors [108]. By evaluating the application of the method in two domains, it was demonstrated that the predictive performance has been improved and the learned model can be used to query oracle selectively to prevent errors. Although a general simulator is needed for flexible learning approaches, control policy in simulation directly applied to robot will yield model errors. In order to overcome cases of severe mismatch, Raileanu et al. proposed a novel way to regularize a decoder of a variational autoencoder to a black-box simulation, with the latent space bound to a subset of simulator parameters [109]. Encoder training from real-world trajectories can yield a latent space with simulation parameter distribution that matches the real setting.

The above methods are mainly to improve the adaptability of state and action in the virtual environment, and try to introduce parameters in the physical environment into the strategy training of the simulated environment [110]. In addition to the three methods mentioned above, there are other ways to improve transfer learning for robot manipulation. Jeong directly introduced the state-related generalized forces to capture the difference between the simulated environment and the real world, thus realizing the transfer learning of robot manipulation [111]. Hwangbo et al. built a perfect actuator model by adding stochastic dynamic parameters, which strengthened the generalization of the neural network [112]. Matas et al. studied the manipulation of non-rigid objects in simulation [113]. Sadeghi et al. studied transfer learning based on multiple domains and proposed a simulation benchmark for robot grasping, which played an important role in promoting the research on robot [114]. Mees et al. proposed an adversarial skill network to find the embedded space suitable for different task domains. This method is not only applicable to the transfer learning of robots, but also to other tasks of finding and learning transferable skills [115].

In summary, methods of transfer learning help us to find out the commonality of problems and deal with the newly encountered problems. The advantage of robot transfer learning lies in learning control strategies based on sufficient data in the simulated environment, while the difficulty of research lies in transferring control strategies to real robots. In the field of robotics, data from simulation can be used to solve problems in which there are few or no sample in the target domain. Dominant approaches for ameliorating transfer learning include building better simulation environment, policy randomization, and direct training of robust policy. Improved methods for robot transfer learning are summarized in Table 3.

## 6. Discussion

The above learning methods can enable robots to make decisions autonomously and adapt various complex environments dynamically. The approach of reinforcement learning generate data from trial-and-error experiments that may damage the robot. Therefore, imitation learning is proposed that robot learns from images, videos, or an expert. Nevertheless, an expert cannot be found anytime, especially when robot manipulation skills are difficult to learn or require extreme precision. In view of this, transfer learning is the appropriate algorithm that train the data in simulation, then the policy refined can be reused on a physical platform [116]. Most notably, robot manipulation leverages the immense progress in learning methods to achieve wonderful developments in many applications. Robot learning application domains can be found in Table 4.

As shown in the above table, the manipulation environment can be classified into three situations, such as industrial robot, personal robot, and medical robot. Previously, robots worked in a structured environment, mainly for delivering, painting, welding, etc. At the same time, they could only perform simple and repetitive tasks with little variation. Currently, robots are gradually able to perform dexterous tasks that ranges from the simple interaction of parts to the complex interaction between humans and the environment. Methods of robot learning address the lack of accurate object models and dynamic changes in complex environments. The learning process is also simplified by visually extracting information from expert presentations [134].

In the training process of deep reinforcement learning, there are two very disturbing problems, namely the design of neural network structure and the setting of hyperparameters. The neural network needs to solve such problems as gradient vanishing, gradient explosion, and overfitting. The appropriate loss functions and activation functions are needed to solve the above two problems. Common loss functions include mean square error, cross entropy error, mean absolute value error, etc. Proper activation functions can make the deep neural network fit the nonlinear model better which mainly include Sigmoid, Tanh, ReLU etc. Moreover, data regularization and dropout are the main methods to solve overfitting. By inserting these processes before the activation function, the deviation of data distribution can be reduced and the accuracy of network can be effectively improved. Neural network architectures need to be experimented and inferred from experimental results. It is recommended to use proven architectures such as VGG, ResNet, Inception, etc. The hyperparameters are the values that initialize the neural network, and these values cannot be learned during training. These super parameters include the number of neural network layers, the size of the batch, the number of trained epochs, etc. Each neural network will have an optimal combination of hyperparameters, which will achieve the maximum accuracy. There is no direct way to get it but usually through trial and error.

It is a challenge to ensure that the learned model is valid, given the interference of the real environment. Much of the collected data are meaningless, so constructing an accurate simulator is hard. Generally, humans solve the new problem via some basic skills. Inspired from this, the method of meta-learning is proposed to generate correct motion sequences that adapts to scene changes based on existing models. Meta-learning is the foundation of both transfer learning and imitation learning that utilizes the previous knowledge and experience to form a core value network [135].

The existing meta-learning neural network structure can be used to accelerate learning when facing new tasks. Model-agnostic meta-learning (MAML) is a meta-learning algorithm for supervised learning and reinforcement learning [136]. The method of MAML makes a back-propagation update of the neural network with the sample, and then completes supervised learning based on the updated parameters. The neural network is forced to learn some task information by adding external data [137,138]. Santoro et al. added external memory to the neural network, which obtained the relevant images for comparison [139]. Marcin et al. trained a general neural network to predict the gradient by the regression problem of equation. As long as the gradient is predicted correctly, this method significantly speeds up the training [140]. Oriol et al. constructed an attention mechanism via imitating humans, which directly focus on the most important parts [141]. Sachin et al. trained an update mechanism of neural networks via the long short-term memory (LSTM) structure, and obtained new parameters by inputting current network parameters [142]. Flood et al. constructed a model to learn and predict the function of loss via previous tasks, which sped up the learning rate [143].

Hence, meta-learning is not a simple mapping, but a way to connect different information. Meta-learning enables the neural network to learn a kind of meta-knowledge based on samples, so that the change factors have been completely separated from the invariant factors in the learned representation space, and the decisive factors can be learned. Although the method has made some progress in the field of robot learning, a large amount of training data is still required in the training phase of meta-learning. Meta-learning is the basis of imitation learning and transfer learning, and one shot learning is an extreme form of the two methods. Therefore, designing a one-shot learning neural network structure with high learning efficiency and excellent performance is an important research direction in the future.

## 7. Conclusions

In view of this method, the robot can understand the intention of samples and map directly to joint control without a lot of training data [144]. With fast learning ability, such a robot system has strong universality. Finn et al. used the visual information to obtain the control information of joints based on the MAML algorithm of Meta-Learning [145]. Tianhe et al. proposed a method of one-shot learning to build prior knowledge by using human and robot demonstration data based on meta-learning. Next, combining this prior knowledge with a person’s video presentation, the robot can perform the tasks demonstrated by the person [146]. Feifei et al. proposed a novel framework of robot learning called neural task programming (NTP), which used neural program induction to do few-shot imitation learning. NTP decomposed the robot’s manipulation into multi-step motions, and the neural network learned how to compose these motions and then execute them. To some extent, it greatly simplifies the difficulty of the problem [147].

To summarize, compared with traditional methods, the methods of robot learning based on deep learning can enable the robot to have the ability of decision-making and learning, which dynamically adapt to many complex situations and greatly improve production efficiency. An end-to-end, completely learned robot with strong imitation learning ability will be the basis for robots to be used in various fields widely. In the future, the complexity of tasks will need to be further increased, such as the one-shot imitation learning in the third person. Improving the efficiency and the generalization in robot learning is also seeking further research attention.

## Figures and Tables

**Table 1:** Robot algorithms based on reinforcement learning.

Model-Based/Free	Ref.	Year	Authors	Algorithm	Value/Policy-Based
	[40]	2010	Hester et al.	DT	Value-based
	[41]	2014	Lioutikov et al.	LLSE	Value-based
Model-based	[42]	2017	Schenck et al.	CNN	Value-based
	[47]	2011	Deisenroth et al.	PILCO	Policy-based
	[50]	2016	Levine et al.	GPS	Policy-based
	[51]	2018	Levine et al.	CEM	Policy-based
	[58]	2015	Zhang et al.	DQN	Value-based
	[63]	2015	Schulman et al.	TRPO	Policy-based
	[33]	2018	Marcin et al.	PPO	Policy-based
Model-free	[64]	2016	Mirowski et al.	A3C	Policy-based
	[66]	2016	Lillicrap et al.	DDPG	Both
	[67]	2016	Gu et al.	NAF	Both
	[68]	2017	Gu et al.	Asynchronous NAF	Both
	[69]	2018	Haarnoja et al.	SAC	Both

**Table 2:** Robot algorithms based on imitation learning.

Categories	Ref.	Year	Authors	Algorithms
	[73]	2007	Takeda et al.	HMM
	[74]	2007	Calinon et al.	GMM
Behavior Cloning	[75]	2010	Calinon et al.	GMR
	[76]	2014	Gams et al.	DMPs
	[77]	2018	Zhang et al.	VL
	[81]	2004	Abbeel et al.	MP
	[82]	2006	Ratliff et al.	MMP
	[83]	2012	Klein et al.	SC
Inverse Reinforcement Learning	[84]	2016	Ho et al.	AL
	[85]	2016	Xia et al.	NIRL
	[87]	2008	Ziebart et al.	Maximum Entropy IRL
	[89]	2011	Boularias et al.	Relative Entropy IRL
	[90]	2018	Peng et al.	DeepMimic
	[94]	2017	Baram et al.	MGAI
	[95]	2017	Merel et al.	Extended GAIL
Generative Adversarial Imitation Learning	[96]	2017	Wang et al.	VAE
	[97]	2017	Stadie et al.	TPIL
	[98]	2017	Liu et al.	IFO

**Table 3:** Improved methods for robot transfer learning.

Improved Methods	Ref.	Year	Authors	Approaches
	[102]	2015	Tzeng et al.	Neural-augmented simulation
Better Simulation	[103]	2017	Zhu et al.	Weak pairwise constraints
	[104]	2018	Peng et al.	Framework of AI2-THOR
	[105]	2014	Ammar et al.	Randomizing the dynamics of the simulator
Policy Randomization	[106]	2016	Rusu et al.	Multi-task policy gradient
	[90]	2018	Peng et al.	Progressive neural networks
	[107]	2018	He et al.	Model-predictive control
Robust Policy	[108]	2020	Ramakrishnan et al.	The oracle feedback
	[109]	2020	Hwasser et al.	Variational auto-regularized alignment

**Table 4:** Robot learning application domains.

Applications	Classical Demos	References
	Peg-in-hole	[117]
Industrial Robot	grinding and polishing	[118]
	welding	[119]
	human-machine collaboration	[120]
	Ironing clothes	[121,122]
Personal Robot	pouring water	[123,124,125]
	autonomous navigation	[53,126]
	obstacle avoidance	[76,127,128]
Medical Robot	Rehabilitation training	[129,130]
	surgical operation	[131,132,133]

## Data Availability

Not applicable.
